# Increasing the Proportion of Broadleaf Species in Mixed Conifer-Broadleaf Forests Improves Understory Plant Composition and Promotes Soil Carbon Fixation

**DOI:** 10.3390/plants14091392

**Published:** 2025-05-05

**Authors:** Zixing Li, Xinghao Wang, Yuan Huang, Xinrong Yang, Ran Wang, Mengtao Zhang

**Affiliations:** College of Forestry, Shanxi Agriculture University, Jinzhong 030801, China; lizixing0927@163.com (Z.L.); wangxing_hao@163.com (X.W.); hyuan04090@163.com (Y.H.); 19834544539@163.com (X.Y.); 18536494038@163.com (R.W.)

**Keywords:** coniferous forests, broadleaf forests, soil properties, soil microbiological composition, understory plant diversity

## Abstract

Understory vegetation is an important component of forest ecosystems, and the supply of nutrients in the soil is related to the growth and development of soil microorganisms and understory plants. The effects of different tree species composition ratios in the forest on the process of soil microbial community assembly are not clear in the existing studies, and the factors influencing the differences in the abundance of understory plants under different forest canopy compositions and their mechanisms of action have not yet been clearly explained. In this study, two types of pure forests (PFP and PFQ) and two types of mixed forests (MF and MPQ) were selected from the Zhongcun Forestry, and the soil characteristics, soil microbial community assembly process, and understory plant community abundance, composition, and β-diversity were analyzed for the different forest types. The results showed that changes in the proportion of broadleaf and coniferous species in the forest could lead to changes in the community assembly process of soil fungi, and that the fungal assembly process in the mixed forest was mainly related to dispersal limitation. Compared with pure forests that were exclusively coniferous or exclusively broadleaf, mixed coniferous and broadleaf forests had a higher abundance of understory plants and a more stable forest community composition. In mixed forests, forests with a large proportion of broadleaf arbors had more available resources in the soil, soil pH was closer to neutral, and soil C was less likely to be lost compared to forests with a large proportion of conifers.

## 1. Introduction

Forests are the most complete and powerful reservoir of natural resources in terrestrial ecosystems [1,2], and northern China has a huge terrestrial biome, whose total area accounts for 11% of the Earth’s land surface [3]. Understory plants are an important component of forest ecosystems and are related to their proper functioning, and the assurance of biodiversity can maintain the spatial and temporal continuity of forest cover [4,5].

Forests cannot grow without soil, and forest soil is an important carbon reservoir in terrestrial ecosystems [6]. Soil microorganisms are the main decomposers of soil organic matter [7], and the supply of nutrients in the soil and soil microorganisms are involved in the soil carbon cycle, linking the above-ground and below-ground parts [8], and maintaining the growth and development of understory plants and the dynamic stability of ecosystem function [9]. It has been shown that different forest canopy compositions lead to differences in the composition and diversity of understory plant communities [10], with understory plants in broadleaf forests exhibiting higher β-diversity than those in coniferous forests at high altitudes [11]. Soil dissolved organic carbon (DOC) is an important factor influencing soil pH, the operation of various nutrients in soil [12], soil microorganisms, and the growth and development of understory plants, and it is one of the most active C pools on land. It has been mentioned in past studies that vegetation restoration can increase the accumulation of unstable soil organic carbon (SOC) [13]. Mixed forests significantly improved soil SOC fixation compared to forests composed of a single species [14], and in temperate climatic zones, mixed forests were more effective at fixing SOC than broadleaf or coniferous forests [15]. It has also been shown that soil DOC can be released to the atmosphere in the form of carbon dioxide [16], and the stabilization of DOC ensures the accumulation of SOC in the soil and reduces the loss of SOC in terrestrial ecosystems, thus enhancing soil carbon sequestration [17]. Changes in soil organic matter content are closely related to the assembly process of soil microorganisms [18], and environmental changes are an important factor influencing the deterministic and stochastic processes of microbial community assembly [19]. In recent years, a large number of studies have focused on the differences arising from the assembly processes of abundant or rare microbial communities in various ecosystems and, to this end, have searched for possible influencing factors. For example, in aquatic ecosystems such as the ocean [20] and lakes [21], enriched microorganisms are more affected by dispersal limitations than rare microorganisms, in contrast to oil-contaminated soils [22], where rare microbial taxa are much more dispersal limited than enriched microbial taxa. However, there is no clear explanation for the assembly process of soil microbial communities in forest ecosystems, and the differences between the assembly processes of soil microbial communities under different tree species mixing methods are not yet clear.

Although these studies have elucidated the important role of mixed forest plantations in soil C fixation, the possible relationship between the differences generated during the assembly of soil microbial communities and changes in soil carbon and understory plant abundance is not clear, whether changes in the ratio of coniferous to broadleaf species in the canopy composition affects understory plant abundance and diversity has not yet been clearly explained, and the mechanism of the role of nutrients in the forest soil under the mixing ratios of different coniferous and broadleaf species is not clear either. In northern China, where there are four distinct seasons and more cold periods, the arbor layer of forests covering the northern region usually consists of both coniferous and broadleaf species [11], and clarifying the relationships between soil properties, changes in soil microbial community assembly processes, and understory plant abundance and diversity in forests with different percentages of coniferous and broadleaf species composition is important for improving soil carbon fixation in forests. This study was undertaken to fill these knowledge gaps and to better identify tree species mixes that increase soil carbon sequestration capacity and enhance understory plant diversity. Based on this, we hypothesized that (i) the proportion of coniferous species mixed with broadleaf species can affect the abundance and diversity of their understory plants; (ii) the proportion of coniferous species mixed with broadleaf species can affect soil carbon sequestration in that soil carbon sequestration capacity is higher in forests with a large proportion of broadleaf species; and (iii) a change in the proportion of coniferous species mixed with broadleaf species can affect the process of assembling soil microbial communities.

## 2. Materials and Methods

### 2.1. Study Area

The study site was situated in Zhongcun Forestry, part of the Zhongtiao Mountain range in Qingshui County, Jincheng City, Shanxi Province, China (111°56′12″–112°14′00″ E, 35°24′00″–35°40′00″ N). This forest lies on the northern slope of Zhongtiao Mountain, a branch of the Taihang Mountains, within a warm-temperate semi-humid continental monsoon climate zone. The forest covers an area of 43,810 hm^2^ with elevations ranging from 1200 to 1700 m above sea level. The region experiences distinct seasonal variations, with an average annual temperature of 10.3 °C and 2679.8 h of sunshine per year. The frost-free period lasts approximately 197 days, and precipitation is concentrated between June and September, averaging 600–800 mm annually. Soil types in the area vary with elevation, transitioning from meadow soil at higher altitudes to brown forest soil, leached brown soil, and finally brown soil at lower elevations (Genetic Soil Classification of China).

China has a wide range of deciduous broad-leaved forests, including the southern part of the north-eastern region and the northern provinces. Because of the cold season, some coniferous species are also planted in these areas to protect against low temperatures and snowfall. Zhongtiao Mountains have the typical artificial mixed coniferous and broad forests of China’s mid-latitude regions. In addition, there are a few pure forests of *Pinus tabuliformis* and *Quercus mongolica*. The zonal vegetation is predominantly deciduous broadleaf forest, and the main vegetation types are *Quercus mongolica*, *Quercus variabilis*, *Populus davidiana*, *Betula platyphylla*, *Carpinus turczaninovii*, *Toxicodendron vernicifluum*, *Pinus tabuliformis,* and *Pinus armandi*, etc.; shrubs mainly include *Forsythia suspensa*, *Rosa xanthina*, *Spiraea trilobata*, and *Prunus davidiana*, etc.; herbs mainly include *Carex lancifolia*, *Themeda triandra*, and *Bothriochloa ischaemum*, etc.

**Figure 1:**
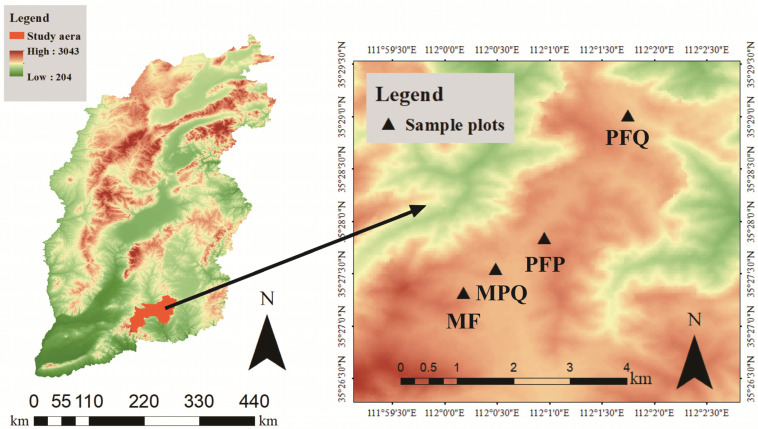
Geographic location and distribution of sample plots.

In July 2024, four different forest types were set up in the Xiachuan Management Area with similar site conditions of Zhongcun Forest. There were two mixed forests: Mixed forests (MF) and Mixed forests of *Pinus tabuliformis* and *Quercus mongolica* (MPQ). There were two types of pure forests: Pure forests of *Pinus tabuliformis* (PFP) and Pure forests of *Quercus mongolica* (PFQ). This totaled four replicates for each forest type. The area of each replicated sample plot was 20 m × 20 m. A total of 16 sample plots (4 × 4) were set up, with a 50 m interval between each two plots. The latitude, longitude, altitude, slope, and aspect of each sample plot were recorded (Figure 1 and Table 1), and the species name, diameter at breast height (DBH), and height of all arbors with DBH ≥ 5 within the sample plot were recorded (Figure S1) and the species composition of each forest type was determined (Figure S2).

### 2.2. Understory Plant Surveys and Soil Sample Collection

In each 20 m × 20 m sample plot, five 5 m × 5 m sample plots were set up according to the five-point sampling method to investigate the species name, height, diameter, and cover of understory arbors and shrubs, and one 1 m × 1 m sample plot was set up in the center of each 5 m × 5 m sample plot to investigate the species name, height, diameter, and cover of understory herbs.

Soil sampling was carried out within each 1 m × 1 m sample plot, with five sampling points in each 20 m × 20 m plot. At each sampling point, three bags of 0–20 cm topsoil were taken, for a total of 15 bags of soil samples per sample plot. After sampling, all soil samples were stored in a portable refrigerator and transported back to the laboratory. After mixing the 15 bags of soil samples from each sample site, large stones and roots were removed with forceps, and then the combined soil samples were sieved through a 0.9 mm sieve. The sieved soil samples from each plot were divided into two equal mass portions; one portion was used for the determination of soil properties and the other portion was used for microbial community composition analysis.

### 2.3. Soil Properties Analysis

Soil pH was measured using a 1:2.5 suspension of soil deionized water [23]. TN (Total nitrogen) was measured using Kjeldahl nitrogen determination [24]. AK (Available potassium) was extracted using 1 M NH_4_Ac [25] and measured using a flame photometer. BD (Bulk density) was determined using the soil core method [26]. SOC (Soil organic carbon) was determined using K_2_Cr_2_O_7_ oxidative wet digestion [27]. DOC (Dissolved organic carbon) extracted with ultrapure water (1:2.5 soil/solution) was quantified using a total organic carbon analyzer [28]. MBC (Microbial biomass carbon) and MBN (Microbial biomass nitrogen) were determined using chloroform fumigation [29,30].

### 2.4. Soil DNA Extraction, Bacterial 16S and Fungal ITS Gene Amplification, and Sequencing

Total DNA was extracted from soil samples using an OMEGA Soil DNA Kit (M5635-02, Omega Bio-Tek, Norcross, GA, USA). The bacterial 16S rRNA V3–V4 region was amplified with primers 338F and 806R [31], while fungal ITS regions were amplified with ITS5 and ITS2 primers [32]. The PCR amplification cycles involved pre-denaturation at 98 °C for 5 min, followed by 25 cycles of denaturation at 98 °C for 30 s, annealing at 52 °C for 30 s, extension at 72 °C for 45 s, and a final extension at 72 °C for 5 min. PCR products were verified using 2% agarose gel electrophoresis, and target fragments were recovered using a magnetic bead recovery method. PCR products were quantified using a BioTek Microplate Reader (FLx800, BioTek Instruments, Winooski, VT, USA) with the Quant-iT PicoGreen dsDNA Assay Kit (Invitrogen, Carlsbad, CA, USA). Sequencing was performed on an Illumina NovaSeq platform with a NovaSeq 6000 SP Reagent Kit (500 cycles, Illumina, San Diego, CA, USA) for 2 × 250 bp paired-end sequencing.

### 2.5. Statistical Analysis

Statistical analyses were performed using Rv4.4.1. A one-way ANOVA followed by Least Significant Difference (LSD) multiple comparisons were applied to analyze significant differences in soil properties among forest types. Data that did not meet normality requirements were transformed using the square root function.

The structure of soil microbial and understory plant communities was analyzed using the Bray–Curtis similarity matrix and principal coordinate analysis (PCoA). Differences in soil microbial and understory plant β-diversity among the four forest types were determined using PERMANOVA with the Adonis function [33]. The phylogenetic composition of soil microbial communities in each sample was determined by calculating the mean nearest taxon distance (MNTD) and nearest taxon index (NTI). MNTD is an abundance-weighted average of the phylogenetic distances between each taxon unit in the sample and its closest relative taxon unit. To assess the extent of non-random phylogenetic community structure, the null distribution of MNTD was obtained by randomizing taxon units and their relative abundance N times in the phylogeny and recalculating the MNTD. NTI quantifies the size of the standardized effect between the observed values of MNTD and the mean of the null distribution. For a single community, a mean NTI > 2 indicates that coexisting taxa are more phylogenetically clustered, and a mean NTI < −2 indicates that coexisting taxa are more phylogenetically over-dispersed [34]. β mean nearest taxon distance (βMNTD) and β nearest taxon index (βNTI) were used to describe the turnover of community phylogenetic composition over space and time (phylogenetic β diversity). βMNTD is the abundance-weighted mean phylogenetic distance between the nearest taxon units in two different communities. The null distribution of βMNTD was obtained by randomizing the taxon units and their relative abundances in the phylogeny N times and recalculating βMNTD. βNTI quantifies the standardized effect size between the observed values of βMNTD and the mean of the null distribution. The assembly process of soil microbial community composition was determined by calculating the β nearest taxon index (βNTI) [35], and the Wilcox test was used to test whether βNTI differed among the four forest types. If |βNTI| > 2.0, the key assembly process of community composition was deterministic (homogeneous selection, βNTI < −2.0; heterogeneous selection, βNTI > 2.0); and if |βNTI| < 2.0, stochastic processes played an important role in microbial community formation [36]. Differences between observed distance values for species taxon and mean values calculated based on the null model were determined using Bray–Curtis based Raup–Crick (RCbary) values. Communities are considered dispersal when |RCbary| > 0.95, homogenizing dispersal for RCbary < −0.95, and dispersal limitation for RCbary > 0.95; when |βNTI| < 2 and |RCbary| < 0.95, the communities were driven by undominated processes [37]. The Kruskal–Wallis test was used to analyze differences in understory arbor and shrub and herb abundance between forest types. RDA analysis was used to investigate the relationship between soil properties, soil microbial NTI, and understory plant abundance, and the significance of marginal and condition effects was determined by the Monte Carlo permutation test [38].

Based on the predictions from the RDA analysis, we used a Partial Least Squares path model (PLS-PM) [39,40] to explore the relationships among understory plant community composition (abundance of all plants in the understory), soil organic carbon (SOC, SOC/DOC), microbial biomass (MBC, MBN/MBC), soil physicochemical properties (pH, BD, AK), TN, and composition of the soil microbial community (F/B NTI). The model is suitable for not normally distributed data. In PLS-PM, there are two sub-models, the external model and the internal model. The external model relates explicit variables to corresponding potential variables, and the internal model assesses the complexity within a system through a network of causal relationships between latent variables. PLS-PM visualizes the results of the path analysis in the form of path coefficients, which are used to test the direction and strength of causal relationships between potential variables, and the external model loadings indicate the extent to which the explicit variables respond to the potential variables [41]. This approach analyzed potential direct and indirect effects among these factors and the abundance of all plants in the understory. In PLS-PM, the direct effects are represented by the corresponding path coefficients, the indirect effects are the paths containing intermediate variables, and the total effect is the sum of the direct and indirect effects describing the relationship between the variables. The fitness of PLS-PM is indicated by the coefficient of determination (R^2^) and the goodness-of-fit (GoF) [40,42]. R^2^ above the cutoff values of 0.19, 0.33, and 0.67 were classified as weak, moderate, and strong, respectively [43]. The GoF above the cutoff values of 0.1, 0.25, and 0.36 were classified as weak, moderate, and strong, respectively [44].

## 3. Results

### 3.1. Soil Characteristics of Different Forest Types

**Figure 2:**
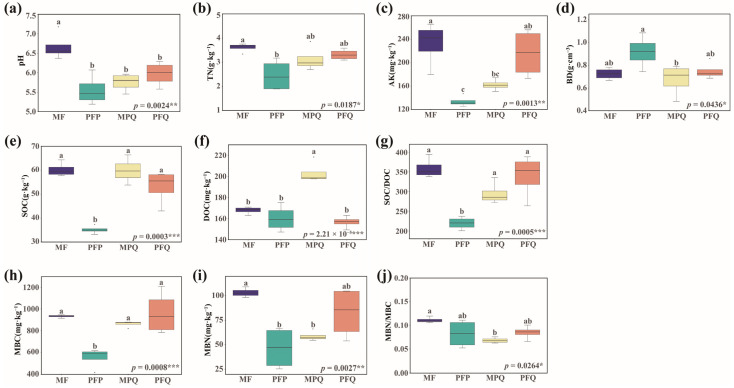
Soil characteristics of different forest types. (**a**) pH, (**b**) total nitrogen (TN), (**c**) available potassium (AK), (**d**) bulk density (BD), (**e**) soil organic carbon (SOC), (**f**) dissolved organic carbon (DOC), (**g**) soil organic carbon/dissolved organic carbon (SOC/DOC), (**h**) microbial biomass carbon (MBC), (**i**) microbial biomass nitrogen (MBN), (**j**) microbial biomass nitrogen/carbon (MBN/MBC). Different letters indicate that this soil property was significantly different (*p* < 0.05) among the four forest types. Asterisks denote significance levels (* *p* < 0.05, ** *p* < 0.01, *** *p* < 0.001).

Soil properties differed significantly (*p* < 0.05) among the four forest types (Table S1). With regard to soil physicochemical properties, the pH of MF was significantly higher (*p* < 0.001) than the other three forest types (Figure 2a) and close to neutral. The TN content was significantly higher (*p* < 0.05) than that of the PFP (Figure 2b). AK was significantly lower (*p* < 0.01) in PFP than in MF and PFQ (Figure 2c). Soil BD was significantly improved by *Pinus tabuliformis* and *Quercus mongolica* mixed, and BD in MPQ was reduced by 27.2% (*p* < 0.05) compared to PFP (Figure 2d). For soil organic carbon, SOC was significantly lower in PFP than in the other three forest types (*p* < 0.001) (Figure 2e), and DOC was the highest in MPQ, which was 21.2%, 26.9%, and 29.7% higher compared with the MF, PFP, and PFQ, respectively (*p* < 0.001) (Figure 2f). For microbial biomass, MBC was significantly lower (*p* < 0.001) in PFP than in the other three forest types (Figure 2g), and MBN content was significantly higher (*p* < 0.01) in MF than in PFP and MPQ (Figure 2h).

### 3.2. Soil Microbial β-Diversity in Different Forest Types

**Figure 3:**
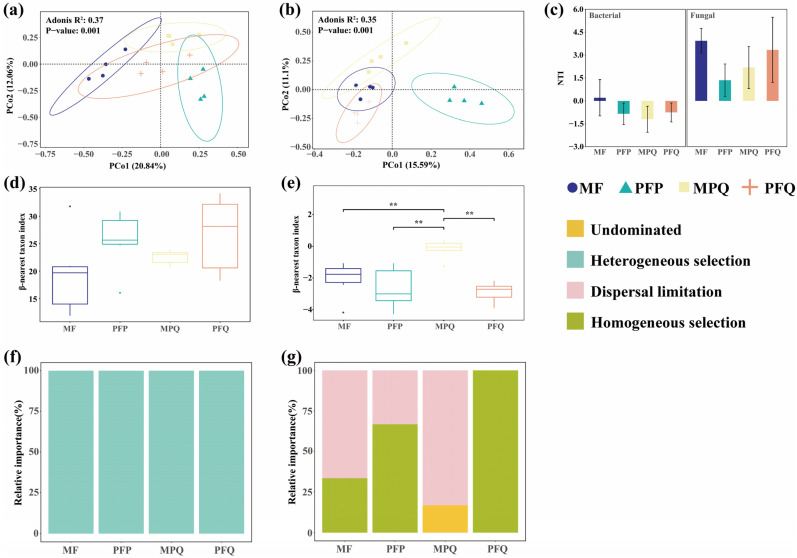
Soil β-diversity in different forest types. (**a**,**b**) PCoA analysis of soil bacteria and fungi, respectively. (**c**) Soil bacterial and fungal NTI in different forest types. (**d**,**e**) Soil bacterial and fungal community assembly processes in different forest types, respectively. (**f**,**g**) Deterministic and stochastic processes in bacterial and fungal community assembly, respectively. Asterisks indicate Wilcox test significance levels (** *p* < 0.01).

β-diversity of soil microbial communities was analyzed at the OTU level using principal coordinate analysis (PCoA) based on the Bray–Curtis distance, and the results showed that the community compositions of soil bacteria (Figure 3a) and fungi (Figure 3b) differed significantly among the forest types. The first two axes of soil bacteria and fungi accounted for 32.9% and 26.69% of the forest type variation, respectively, and the fungal community composition of PFP was significantly separated from the other three forest types.

The mean NTI of bacteria in MF was the largest among the four forest types and greater than 0; the mean NTI of bacteria was lower than 0 in each of the other three forest types. The mean NTI of fungi in each of the four forest types was greater than 0, and the mean NTI of fungi in each of the three forest types was greater than 2, except for PFP (Figure 3c). The βNTI values of the bacteria were all >2.0, indicating that deterministic processes regulate the assembly of bacterial communities in the four forest types, and that the aggregation of bacterial communities in four forest types was driven by heterogeneous selection processes (Figure 3d,f). The βNTI value of PFQ fungi was <−2.0, indicating that the assembly of fungal communities in this forest type was regulated by deterministic processes. Among the four forest types, the aggregation of fungi in PFQ showed the highest homogeneous selection, followed by that in PFP. The aggregation of fungi in MF and MPQ was mainly driven by dispersal limitation, and the aggregation of fungi in MPQ was also driven by undominated processes, and the βNTI value of fungi in MPQ was significantly higher than the βNTI value in the other three forest types (*p* < 0.01), with stochasticity processes regulating the assembly of their fungal communities (Figure 3e,g).

### 3.3. Understory Plant Composition and Diversity in Different Forest Types

The survey showed that a total of seven families of understory arbors, 12 families of shrubs, and 15 families of herbs were recorded within the four sample plots of MF. In the four sample plots of PFP, a total of 11 families of understory arbors, 10 families of shrubs, and 14 families of herbs were recorded. A total of nine families of understory arbors, eight families of shrubs, and 11 families of herbs were recorded in the four sampling plots of MPQ. In the four sample plots of PFQ, a total of 12 families of arbors, nine families of shrubs, and 16 families of herbs were recorded in the understory.

**Figure 4:**
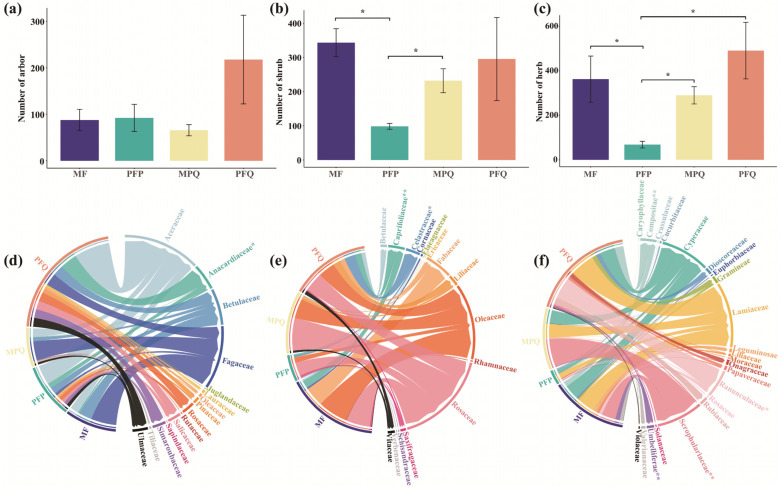
Understory plant composition of different forest types. (**a**–**c**) Comparison of the number of arbors, shrubs, and herbs in the understory of four forest types, respectively. (**d**–**f**) Understory arbor, shrub, and herb composition of four forest types under family classification, respectively. Asterisks indicate the significance level of the Kruskal–Wallis test (* *p* < 0.05, ** *p* < 0.01).

At the family level, the Kruskal–Wallis test showed that there was no significant difference in the number of understory arbors among the four forest types (Figure 4a), the number of understory shrubs was significantly lower (*p* < 0.05) in PFP than in MF and MPQ (Figure 4b), and the number of understory herbs in PFP was significantly lower (*p* < 0.05) than that of the other three forest types (Figure 4c). Among the four forest types, the three dominant understory arbors belonged to Aceraceae, Fagaceae, and Betulaceae, and the number of understory arbors of Aceraceae was lower in mixed forests (MF, MPQ) than in pure forests (PFP, PFQ) (Figure 4d and Table S2); the dominant and the top three understory shrubs belonged to Rosaceae, Oleaceae, and Fabaceae and the numbers of the three shrubs in PFP were the smallest among the four forest types (Figure 4e and Table S3); the dominant and top three understory herbs belonged to Lamiaceae, Cyperaceae, and Scrophulariaceae, and the number of Scrophulariaceae herbs differed significantly (*p* < 0.01) among the four forest types (Figure 4f and Table S4).

**Figure 5:**
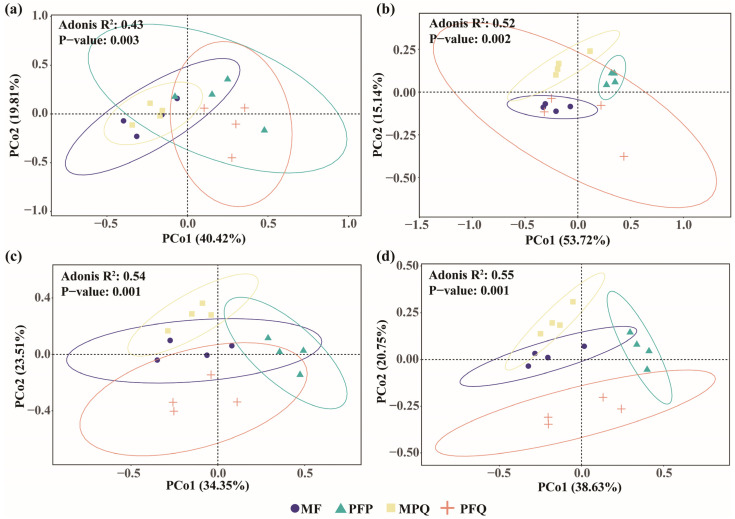
β-diversity of understory plants in different forest types. (**a**–**c**) PCoA analysis of understory arbors, shrubs, and herbs, respectively. (**d**) PCoA analysis of all plants in the understory.

β-diversity of understory plant communities was analyzed at the family level using principal coordinate analysis (PCoA) based on the Bray–Curtis distance, and the results showed that the community composition of understory arbors (Figure 5a), shrubs (Figure 5b), herbs (Figure 5c), and all plants (Figure 5d) differed significantly among forest types. The first two axes of understory arbors, shrubs, and herbs accounted for 60.23%, 68.86%, and 57.86% of the forest type variation, respectively; the first two axes of understory all plants accounted for 59.38% of the forest type variation.

### 3.4. Influence of Soil Properties and Microbial Diversity on the Composition of Understory Plants

**Figure 6:**
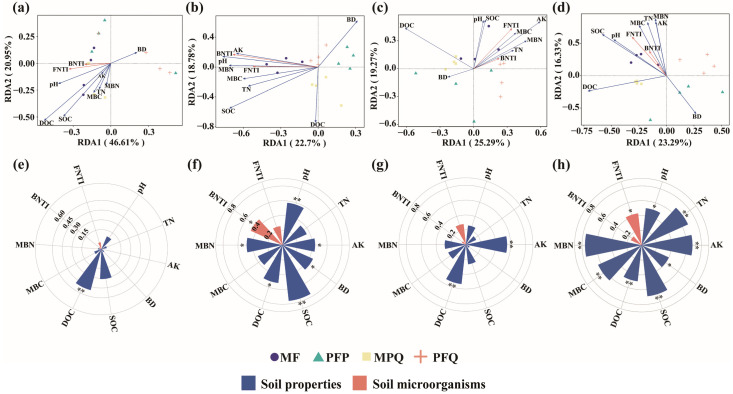
Redundancy analysis (RDA) of soil properties, soil microorganisms and understory plants abundance. (**a**–**c**) RDA analysis of understory arbors, shrubs and herbs abundance, respectively. (**d**) RDA analysis of all plants abundance in the understory. (**e**–**h**) The contributions of environmental factors to changes in the composition of the understory arbors, shrubs, herbs and all plants, respectively. Asterisks denote significance levels (* *p* < 0.05, ** *p* < 0.01).

RDA analyses showed that soil properties and soil microorganisms were closely related to changes in the abundance of understory plants. The first two RDA axes explained 67.56%, 41.48%, 44.56%, and 39.62% of the total variation in the community composition of understory arbors (Figure 6a), shrubs (Figure 6b), herbs (Figure 6c), and all plants (Figure 6d). DOC (*p* < 0.01) was significantly correlated with changes in the composition of the understory arbor community (Figure 6e); pH (*p* < 0.01), AK (*p* < 0.05), BD (*p* < 0.05), SOC (*p* < 0.01), DOC (*p* < 0.05), MBN (*p* < 0.05), and bacterial NTI (*p* < 0.05) were significantly associated with changes in understory shrub community composition (Figure 6f); AK (*p* < 0.01) and DOC (*p* < 0.01) were significantly associated with changes in understory herb community composition (Figure 6g); pH (*p* < 0.05), TN (*p* < 0.01), AK (*p* < 0.01), BD (*p* < 0.05), SOC (*p* < 0.01), DOC (*p* < 0.01), MBC (*p* < 0.01), MBN (*p* < 0.01), and fungal NTI (*p* < 0.05) were significantly correlated with changes in the composition of all plant communities in the understory (Figure 6h and Table S5).

**Figure 7:**
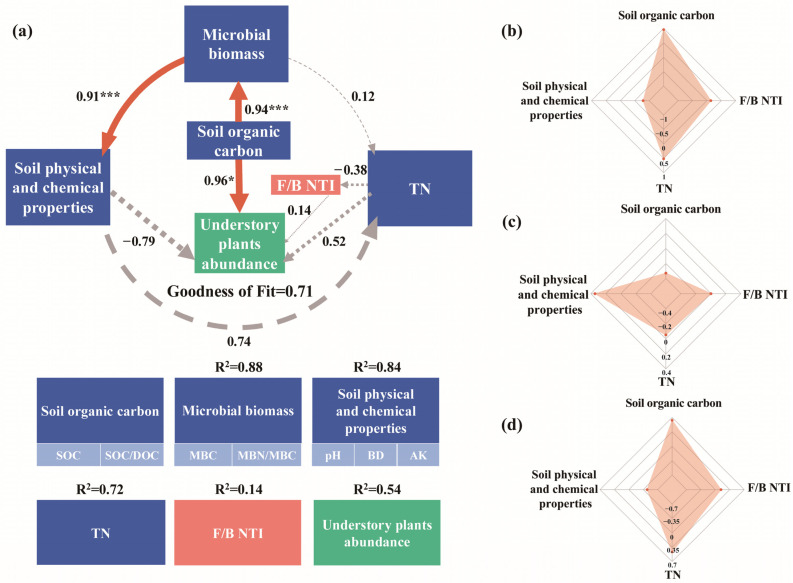
Relationships between soil organic carbon, microbial biomass, soil physical and chemical properties, TN, F/B NTI, and understory plants abundance. (**a**) The PLS-PM plot of the effects of soil organic carbon, microbial biomass, soil physical and chemical properties, TN, and F/B NTI on understory plants abundance. Orange solid arrows indicate significant positive effects; gray dashed arrows indicate non-significant effects. This model was evaluated using the goodness-of-fit (GoF) statistic, an indicator of overall predictive performance. For the PLS-PM represented here, the GoF was 0.71. The standardized direct (**b**), indirect (**c**), total (**d**) effect of each predictor on understory plants abundance from the PLS-PM used. Asterisks denote significance levels (* *p* < 0.05, *** *p* < 0.001).

In addition, the Partial Least Squares path model (PLS-PM) integrated the complex interrelationships among soil organic carbon, microbial biomass, soil physical and chemical properties, TN, soil microbial community composition, and understory plant community composition. The GoF of the model was 0.71. PLS-PM results showed that soil organic carbon (SOC, SOC/DOC) had a significant positive effect (r = 0.94, *p* < 0.001) on microbial biomass (MBC, MBN/MBC) and a significant positive effect (r = 0.95, *p* < 0.05) on understory plant community composition (abundance of all plants in the understory); Microbial biomass (MBC, MBN/MBC) had a significant positive effect (r = 0.91, *p* < 0.001) on soil physical and chemical properties (pH, BD, AK) (Figure 6a). Soil organic carbon (SOC, SOC/DOC), TN, and soil microbial community composition (F/B NTI) directly influenced understory plant community composition (abundance of all plants in the understory), and soil physicochemical properties (pH, BD, AK) indirectly influenced understory plant community composition (abundance of all plants in the understory) (Figure 7).

## 4. Discussion

### 4.1. Effects of Different Forest Types on the β-Diversity of Soil Microorganisms

Soil bacterial and fungal β-diversity differed significantly among the four forest types, with a stronger effect of forest type on soil microbial community β-diversity. The results of the NTI calculations showed that the mean NTI values of bacteria in PFP, MPQ, and PFQ were all less than 0. The mean NTI of bacteria in MPQ was lower than that in PFP and PFQ, indicating that the soil bacteria in MPQ were more phylogenetically over-dispersed than those in the remaining two pure forests [35]. The results of βNTI calculations indicated that deterministic processes drove bacterial community assembly, that bacterial community assembly was dominated by heterogeneous selection, and that bacterial phylogenetic turnover in the four forest types exceeded expectations. Compared to pure forests, the community assembly processes of fungi in mixed forests were all deterministic rather than stochastic. Fungal communities in PFQ were dominated by homogeneous selection, and due to strong homogeneous selection, fungal communities occupied narrower environmental ecological niches, and fungal community phylogenetic turnover was less than expected [35]. Fungal assembly processes in MF and MPQ have been associated with dispersal limitation, with very limited dispersal between soil communities in mixed forests [36], and also with non-dominated stochastic processes, probably because stochastic processes can play a role in high resource availability, and the relatively high SOC content of MPQ provides a rich source of available resources for the assembly processes of their soil fungi [18,45].

### 4.2. Effects of Different Forest Types on Understory Plant Abundance and β-Diversity

Among the four forest types, the abundance of understory plants was lowest in PFP, and the top three species ranked in the abundance of shrubs and herbs in its understory were lower than those in the other three forest types. PCoA analyses showed that the effects of forest type on the β-diversity of understory communities were stronger, and the differences in the β-diversity of arbors, shrubs, herbs, and all plants in the understory of the four forest types were significant. The main source of these differences was the difference in canopy species composition [46,47], where different species compositions can provide different seed sources for the forest, and forest types with rich canopy compositions had rich seed sources and their understory plant compositions were richer compared to those with a single canopy composition. The PFP, characterized by tall and straight trunks, single-species composition, and minimal variation among individuals, has long maintained a monotonous environmental state, which has significantly restricted the diversity of understory plants [48]. However, the understory plant diversity of PFQ with a single canopy composition was significantly higher than that of PFP, and even higher than that of the two mixed forests, which does not disprove the conclusion that the understory species composition of forests with a single canopy composition is homogeneous, because the understory species diversity of the forests with broadleaf species in the canopy group was higher than that of the forests with coniferous forests in the canopy group [11,49]. Among the four forest types, the canopy composition in PFQ was all broadleaf species, the canopy species in PFP was all coniferous species, and the coniferous species in MF was slightly less than that in MPQ, so its understory plant abundance showed the result that MF was closer to PFQ.

### 4.3. Relationships Between Soil Properties, Soil Microorganisms, and Understory Plant Community Composition

There is a close relationship between soil properties, soil microbial β-diversity, and understory plant community abundance. DOC content was the main factor affecting the abundance of understory arbors and understory herbs, and DOC content was significantly higher in MPQ than in the other three forest types, and in addition to serving as an important carbon source for soil microbial growth and development [50], the higher DOC content contributed to the stabilization of SOC [6]. Since DOC is easily oxidized to CO_2_ or dissolved in water [51], a low proportion of DOC in organic carbon represents CO_2_ that is less likely to be released to the atmosphere and soil C that is less likely to be lost. SOC/DOC was significantly lower in PFP than in the other three forest types, and the large amount of C loss in PFP was detrimental to the growth of understory plants. pH and SOC content were the main factors influencing the abundance of understory shrubs. The abundance of understory shrubs in MF was the highest among the four forest types, and the abundance of understory shrubs in PFP was the lowest among the four forest types. This is because organic acid leaching during pine needle decomposition leads to soil acidification [52], and among the four forest types, MF had the lowest planting of *Pinus tabuliformis*, which had less acidic soil pH compared to PFP, which was full of *Pinus tabuliformis*. Changes in soil pH were also influenced by soil SOC content, with lower SOC content making it difficult to release large amounts of H^+^ ions from the soil and enhancing soil acidity [53]. Higher SOC content in MF resulted in more H^+^ ions being released from the soil, and the soil pH was close to neutral, which was more suitable for the growth of understory shrubs. In addition, MF, MPQ, and PFQ with more broadleaf species had higher soil pH (less acidic) and TN content than pure coniferous forest (PFP) [54], and the abundant Fabaceae shrubs with nitrogen-fixing capacity in the understory of MF were also responsible for the significantly higher soil TN and MBN content than in the other three forest types [55].

Overall, organic carbon was the most important factor affecting the abundance of all plants in the understory; this is consistent with previous findings. A previous study showed a significant increase in carbon sequestration capacity in mixed forests compared to pure forests of a single species [14]. PLS-PM results showed that organic carbon directly affected the abundance of all plants in the understory, in addition to being an important predictor of soil microbial biomass. This is because, in mixed forests, increasing the proportion of broadleaf species in mixed forests ensures a diversity of plant functions in the forest, a wider range of apomictic input types, more SOC accumulation, and a diverse composition of soil microbial communities [15]. The abundant SOC provided sufficient nutrients for the growth of understory plants, and the diverse microbial composition improved the microbial biomass in the soil. Plants need to absorb more fast-acting nutrients for photosynthesis during growth and development [45,56], lower AK content will limit the growth of understory plants, and the lower AK content of PFP led to its lower understory plant abundance. The BD of PFP was the highest among the four forest types, and lower oxygen availability in a high BD environment would make it difficult for a large number of aerobic microorganisms to survive and reproduce [50], while the F/B NTI directly affected all plant abundance in the understory, suggesting that the closeness of coexisting taxa within the soil microbial community predicts understory plant abundance, thus explaining the correlation that exists between the BD and the understory plant abundance. Together, these results suggest that increasing the proportion of broadleaf species in mixed forests is an important way to improve the sustainability of forest ecosystems. This study identified that planting more broadleaf species in mixed forests is a more effective forest management measure to improve soil nutrients and enhance ecosystem stability than planting a purposeless mixture. As the study area is located in the warm-temperate continental climate zone, the tree species mixes suitable for other climate types are not yet known, and the scope of the survey should be expanded to compare the differences in tree species mixes in different climate zones and the applicability of the forest management initiative of expanding the proportion of broadleaf species planted in other climatic zones.

## 5. Conclusions

This study revealed the factors influencing the differences in understory plant abundance in forests of different forest types by analyzing the relationships between soil properties, soil microorganisms, and understory plants in four forest types. Overall, forests with a large proportion of broadleaf arbors had more available resources in the soil and higher understory plant abundance compared to forests with a large proportion of coniferous arbors. More broadleaf arbors in a forest can improve soil pH and fix more carbon to provide essential nutrients for understory plants to grow and develop, and a change in the proportion of broadleaf and conifer species in forests can also lead to a change in the process of community assembly of soil fungi. Increasing the proportion of broadleaf species in mixed coniferous-broad forest will increase the diversity of understory plants, and at the same time, it will regulate the nutrient content of forest soils and change the whole forest towards a richer diversity of species. The study provides a theoretical basis for forest management.

## Figures and Tables

**Table 1:** Basic conditions of sample plots in different forest types. MAT indicates mean annual temperature. MAP indicates mean annual precipitation.

Sample Plot Type	Dominant Tree Species	Longitude	Latitude	Altitude/m	Slope/°	Aspect	MAT/°C	MAP/mm
MF	*Pinus tabuliformis*, *Quercus mongolica*, *Populus davidiana*, *Betula platyphylla*, *Carpinus turczaninovii*	112°0′10″	35°27′19″	1747	15	E	6.9	753
PFP	*Pinus tabuliformis*	112°0′57″	35°27′50″	1613	19	N	7.4	736
MPQ	*Pinus tabuliformis*, *Quercus mongolica*	112°0′29″	35°27′32″	1675	27	S	7.1	747
PFQ	*Quercus mongolica*	112°1′44″	35°29′1″	1664	23	E	7.3	702

## Data Availability

Data will be made available on request.

## Supplementary Materials

The following supporting information can be downloaded at: https://www.mdpi.com/article/10.3390/plants14091392/s1, Figure S1: Basic conditions of arbors in the sample plots of different forest types. (a) Mean DBH, (b) Mean height, (c) Stand density. Figure S2: Proportion of community composition of coniferous and broadleaf species in different forest types. Table S1: One-way ANOVA for soil properties in different forest types. Table S2: Number of arbors in the understory of different forest types under family classification. Table S3: Number of shrubs in the understory of different forest types under family classification. Table S4: Number of herbs in the understory of different forest types under family classification. Table S5: Contribution of environmental factors to changes in understory plant composition.
