# Supplementary material for: Increasing the Proportion of Broadleaf Species in Mixed Conifer-Broadleaf Forests Improves Understory Plant Composition and Promotes Soil Carbon Fixation
Source: Plants (Basel). 2025 May 5;14(9):1392. doi: 10.3390/plants14091392 (PMC12073394; doi:10.3390/plants14091392)
Supplement: Supplementary file 1 [file plants-14-01392-s001.zip › plants-3597618-supplementary.pdf]

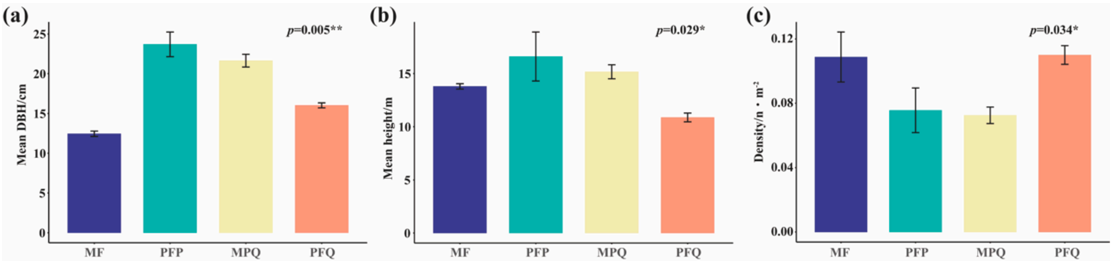

Figure S1. Basic conditions of arbors in the sample plots of different forest types. (a) Mean DBH, (b) Mean height, (c) Stand density.

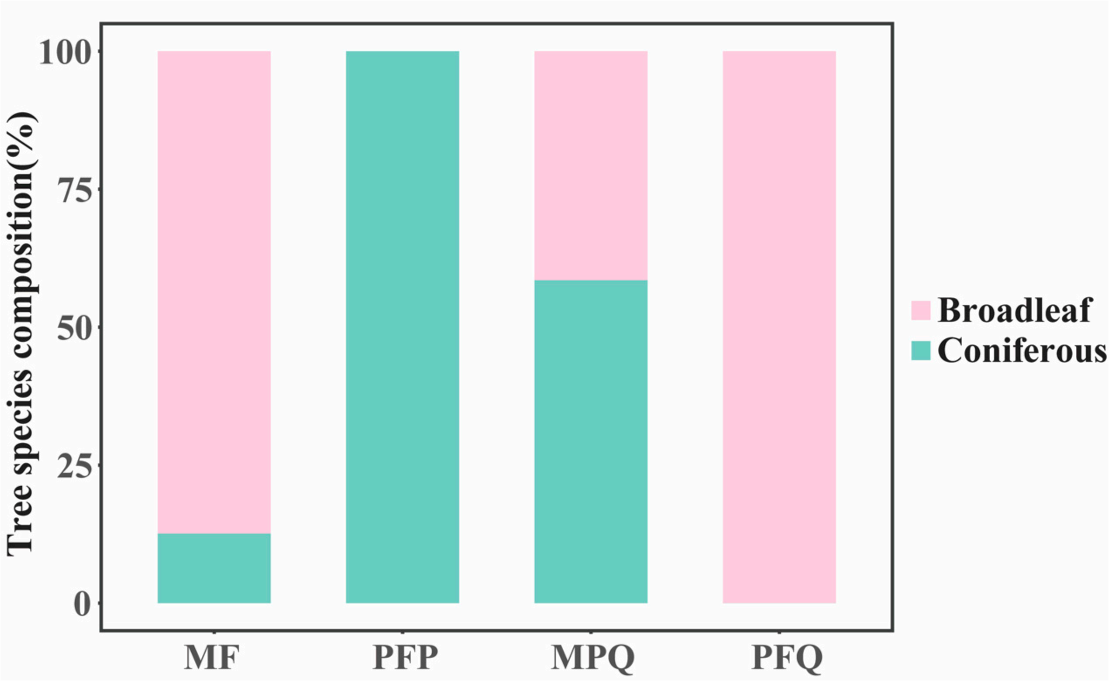

Figure S2. Proportion of community composition of coniferous and broadleaf species in different forest types.

Table S1. One-way ANOVA for soil properties in different forest types.

	Soil characteristics	ANOVA（*p*-value）
Soil physical and chemical properties	pH	**0.0024** **
	TN($g·kg^{-1}$)	**0.0187** *
	AK($mg·kg^{-1}$)	**0.0013** **
	BD($g·cm^{-3}$)	**0.0436** *
Soil organic carbon	SOC($g·kg^{-1}$)	**0.0003** ***
	DOC($mg·kg^{-1}$)	**2.21e-05** ***
	SOC/DOC	**0.0005*** **
Microbial biomass	MBC($mg·kg^{-1}$)	**0.0008** ***
	MBN($mg·kg^{-1}$)	**0.0027** **
	MBN/MBC	**0.0264** *

Note: Asterisks denote significance levels (*$p < 0.05$, **$p < 0.01$, ***$p < 0.001$)

Table S2. Number of arbors in the understory of different forest types under family classification.

Family	MF	PFP	MPQ	PFQ
Aceraceae	62	143	63	258
Anacardiaceae	12	38	3	125
Betulaceae	94	49	26	77
Fagaceae	183	19	139	89
Juglandaceae	0	0	0	3
Lauraceae	0	6	0	14
Oleaceae	0	6	0	17
Pinaceae	0	12	0	31
Rosaceae	0	24	0	62
Rutaceae	0	0	1	2
Salicaceae	0	24	1	64
Sapindaceae	0	0	1	0
Simaroubaceae	0	24	2	64
Tiliaceae	0	0	12	0
Ulmaceae	0	24	15	64

Table S3. Number of shrubs in the understory of different forest types under family classification.

Family	MF	PFP	MPQ	PFQ
Betulaceae	24	0	3	64
Caprifoliaceae	120	20	0	102
Celastraceae	5	30	3	173
Cornaceae	23	0	0	0
Elaeagnaceae	0	0	3	0
Ericaceae	1	1	0	0
Fabaceae	308	0	25	75
Liliaceae	0	4	38	118
Oleaceae	469	79	309	264
Rhamnaceae	11	0	0	0
Rosaceae	373	214	513	307
Saxifragaceae	27	31	0	22
Schisandraceae	2	4	0	0
Verbenaceae	0	10	0	0
Vitaceae	9	1	33	57

Table S4. Number of herbs in the understory of different forest types under family classification.

Family	MF	PFP	MPQ	PFQ
Caryophyllaceae	0	2	0	0
Compositae	105	5	15	174
Crassulaceae	0	4	0	0
Cucurbitaceae	0	22	0	7
Cyperaceae	291	70	260	302
Dioscoreaceae	27	6	3	89
Euphorbiaceae	7	0	4	0
Gramineae	34	6	7	89
Lamiaceae	359	104	274	487
Leguminosae	65	0	4	0
Liliaceae	29	3	0	13
Moraceae	0	4	0	6
Onagraceae	4	7	0	80
Papaveraceae	23	0	0	54
Ranunculaceae	61	4	16	441
Rosaceae	0	18	0	0
Rubiaceae	0	14	0	133
Scrophulariaceae	283	0	565	28
Solanaceae	0	0	0	15
Umbelliferae	90	5	9	29
Valerianaceae	69	0	3	3
Violaceae	3	0	0	13

Table S5. Contribution of environmental factors to changes in understory plant composition.

	Arbor		Shrub		Herb		All understory plants	
	r^2	*p*	r^2	*p*	r^2	*p*	r^2	*p*
pH	0.14	0.332	0.58	**0.004****	0.26	0.146	0.50	**0.011***
TN	0.06	0.703	0.36	0.064	0.15	0.335	0.68	**0.002****
AK	0.01	0.916	0.44	**0.026***	0.56	**0.004****	0.68	**0.001****
BD	0.04	0.805	0.45	**0.023***	0.05	0.691	0.40	**0.028***
SOC	0.31	0.086	0.76	**0.001****	0.27	0.136	0.69	**0.002****
DOC	0.44	**0.033***	0.52	**0.011***	0.54	**0.005****	0.49	**0.009****
MBC	0.08	0.608	0.36	0.058	0.26	0.131	0.64	**0.002****
MBN	0.04	0.764	0.48	**0.018***	0.28	0.097	0.76	**0.001****
BNTI	0.03	0.853	0.47	**0.013***	0.06	0.700	0.16	0.325
FNTI	0.07	0.589	0.26	0.123	0.28	0.094	0.43	**0.027***

Note: Significant *p* values ($p < 0.05$) were indicated in bold. Asterisks denote significance levels (**p* < 0.05, ***p* < 0.01, ****p* < 0.001)
